# Knowledge, and use of labour pain relief methods and associated factors among obstetric caregivers at public health centers of East Gojjam zone, Amhara region, Ethiopia: a facility based cross- sectional study

**DOI:** 10.1186/s12884-020-2844-z

**Published:** 2020-03-23

**Authors:** Keralem Anteneh Bishaw, Endalew Gemechu Sendo, Workinesh Sinshaw Abebe

**Affiliations:** 1 Department of Midwifery, Debre-Markos University, College of Medicine and Health Sciences, Debre-Markos, Ethiopia; 2 School of Nursing and Midwifery, Addis-Ababa University, College of Health Science, Addis Ababa, Ethiopia

**Keywords:** Knowledge, Labour pain relief methods, Obstetric caregivers, Ethiopia

## Abstract

**Background:**

The study was conducted in public health centers of East Gojjam Zone, Amhara region, Ethiopia**.** The purpose of this study was to assess knowledge, and use of labour pain relief methods and associated factors among obstetric caregivers in the study setting.

**Methods:**

A facility-based cross-sectional study design was conducted from March 1–30, 2018. The study was conducted among three hundred and nine sampled obstetric caregivers. Structured questionnaire was used to collect the data. The data were entered into Epi-data version 4.2 Software for cleaning and exported to SPSS version 23.0 for data analysis. Multivariate logistic regression was carried out for variables with a *p*-value < 0.25 in bivariate logistic regression to determine significant relationships between the dependent and independent variables. Statistical significance was determined at 95% confidence interval (CI) and *p*-value below 0.05.

**Result:**

The overall use of labour pain relief methods reported was 34.4%, (30.4% non-pharmacological and 8.4 pharmacological, respectively). More than half of the study participants (54.2%) had adequate knowledge about labour pain relief methods. In multivariate analysis, being a midwifery profession [AOR =2.814, 95% CI = (1.574–5.031)], having positive attitude [AOR = 4.370, 95% CI = (2.523–7.567)], and professionals with a medium level of education [AOR = 3.450, 95% CI = (1.993–5.971)] were factors significantly associated with knowledge of obstetric caregivers about labour pain relief methods. In multivariate analysis, knowledge of obstetric caregivers [AOR = 3.821, 95% CI = (2.091–6.980)], positive attitude of obstetric caregivers [AOR = 2.455, 95% CI = ((1.358–4.436))] and experience of obstetric caregivers [AOR = 2.56, 95% CI = (1.350–4.845) were factors significantly associated with the use of labour pain relief methods.

**Conclusion:**

In this study, the overall use of labour pain relief methods by obstetric caregivers was low. Systemic opioid (Pethdine) was reportedly one of the most known pharmacological pain relief methods in this study. Providers’ knowledge, attitude and work experience had shown statistical significance with the use of labour pain relief methods. Task-oriented in- service training is required to fortify obstetric caregivers’ knowledge and attitude towards the use of labour pain relief methods.

## Background

Pain accompanies a human being since the beginning of his or her existence and is widely recognized as a negative phenomenon. In fact, it is an indispensable element of our life. It signals the worsening of health (called pathologic pain in that case), or it supports the progress of natural processes taking place in the body, e.g. during labour (physiological pain) [1].

Since creation, human beings have constantly felt pain and have always tried to control it in different ways. Labour pain is one of the most severe pains. This pain, as one of the inevitable aspects of the childbirth process, is unlike from other pains. It is not a sign of injury or (tissue damage), reduces spontaneously, is regular and continuous, gets tense gradually, and leads to a pleasant incident, which is childbirth [2].

Pain during labour is a central part of women’s experience of childbirth, as its excruciating nature makes most women want to alleviate it. Childbirth is among one of the most intense pain that majority of women will endure during their lifetime [3, 4]. Unresolved past psychological or physical distress along with loneness, lack of knowledge, unfriendly or unresponsive treatment during labour might surge the chance that the woman will suffer. In sub-Saharan Africa particularly in Nigeria, giving birth may not be an excited incident, however, it can be an unhappiness experience owing to some midwives’ attitude towards the laboring woman who scream at labouring woman mainly when she cries or complains of labour pain [5].

Various pharmacologic and non-pharmacologic treatments have been developed to alleviate the labour pains; and their use has become popular, specifically in developed countries [6]. Pain relief during labour is desired by many women, irrespective of race or belief, and contributes enormously to their satisfaction with the experience of childbirth. Labour pain can be perceived to be the most severe form of pain experienced in a woman’s life. Studies have shown that when women are offered analgesia during labour, they report greater satisfaction with their overall birth experience [7, 8].

A study in Southeast Nigeria among Igbo women reported 67.6% of labouring women need labor pain alleviation, however, only 27% of parturient received pain relief during labour [9]. Another study in Aga Khan teaching and referral hospital in Kenya found that 90% of woman would request some form of labour pain relief at their next delivery but 18% had been offered some form of pain relief at their last delivery with 82% of those offered having effective pain relief as reported by the study participants [10].

Although labor pain management is accepted and implemented in many countries of the world, in Ethiopia pain management during labor is not a common practice. This might be as a result of a number of factors: the availability of drugs, health care delivery systems, limited knowledge, providers’ attitude about labor pain management, and choice of caregivers and clients. Of these, the attitude, knowledge, and skills of the provider to offer labour analgesia are vital, particularly in low-income countries, including Ethiopia [11, 12]. Therefore, this study aimed at assessing the level of knowledge, use of labour pain relief methods and associated factors among obstetric caregivers at public health centers of East Gojjam Zone, Amhara Region, Ethiopia.

## Methods

### Study design

A facility-based cross-sectional study was conducted.

### Study area and period

The study was conducted in public health centers of East Gojjam Zone from March 1–30, 2018. East Gojjam is one of an administrative zone in Amhara regional state of Ethiopia. Debre Markos town is the capital city of East Gojjam zone, which is 265 Km far from Bahirdar, the capital city of Amhara region and 299 Km from Addis Ababa, the capital city of Ethiopia. According to 2010 Health Bureau of East Gojjam Zone, there were 100 public health centers, and 329 health officers, 797 nurses and 307 midwives working in the zone (district).

### Sample size, population and sampling technique

We selected thirty-three (33) public health centers (33%) out of 100 public health centers located in the study area by simple random sampling technique (lottery method). The source population of the study was health professionals in public health centers of East Gojjam Zone, Amhara region, Ethiopia. The study population was obstetric caregivers available during the study period in sampled health centers. Three hundred and nine (309) sampled obstetric caregivers; (including midwives, nurses, and health officers) who were giving obstetric care in the delivery room were consented and included in the study. Health care providers came to labour ward for consultation during study period were excluded from the study. Since there were small numbers of study population in the study area, all obstetric care caregivers, who were available during the study period were considered as study participants.

### Data collection tools and procedures

A Structured pretested self-administered questionnaire was used to collect the data. The questionnaire was adapted from reviewed literature [13–15], and with some amendments into the local context. The questionnaire consisted of seven parts: the first part was used to assess socio-demographic characteristics of obstetric caregivers while the rest were used to assess the knowledge, attitude, use, preference of labour pain relief methods and institutional factors affecting the use of labour pain relief method. The questionnaire was designed in English to be understood by every study participants. Nine diploma nurses were recruited for data collection and two BSc midwives were hired for the supervision of data collection procedure.

### Measurement

Knowledge about labour pain relief methods were measured by a 10-item knowledge questionnaire adapted from previous studies [13–15]. The scale for assessing knowledge were from 0 to 10 scores. Correct answers were given a score of 1 and incorrect answers 0. Those who scored less than the mean value were considered to have inadequate knowledge while those who scored greater than or equal to the mean value were considered as having good knowledge.

Use of labor pain relief method was measured by the following question: Obstetric care provider who answered “yes” for the question “Have you ever provide any labor pain relief method in the past one month? Attitude towards labor pain relief method: A seven (7) item response options (Yes/No) were adapted from prior studies [13, 14]. The total score were computed for each respondent and it ranges from 0 to 14 scores. Those who score less than the mean value were considered to have negative attitude while those who score greater than or equal to the mean value were considered as having positive attitude towards labour pain relief methods.

### Data quality control

Training was provided for data collectors and supervisors on objective, the benefit of the study, individual’s right and informed consent for the common understanding of the study in general and the questionnaire in particular. A pre - test was done in west Gojjam zone public health centers on 5% of obstetric caregivers two weeks before the actual data collection time. Regular supervision during data collection was made; the questionnaire was reviewed and checked for completeness, accuracy and consistency by the research team and supervisors.

### Data analysis

First, the questionnaire was checked for completeness. The data were entered into Epi-data version 4.2 Software for cleaning and exported to SPSS version 23.0 for data analysis. Descriptive statistics were computed to determine frequencies and summary statistics (mean, standard deviation, and percentage) to describe the study population in relation to socio-demographic and other related variables. Bivariate logistic regression was carried out to see the association of each of the independent variables with the outcome variable. Multivariate logistic regression was then carried out for variables with a *p*-value < 0.25 to determine significant relationships between the dependent and independent variables. Statistical significance was determined at 95% confidence interval (CI) and *p*-value below 0.05.

## Results

### Socio demographic characteristics of respondents

Out of the 309 sampled obstetric caregivers, 299 responded to the questionnaires making a response rate of 96.8%. The mean age of the respondents was 28.96 (± SD = 4.195) years. A significant number 194 (64.9%) of them were in the age group of 20–29 years. More than half 162 (54.2%) of the respondents were males and the majority (86.6%) were Orthodox Christians. Out of the total respondents, 31.1% were midwives by their profession. Nearly half 149 (49.8%) of study participants were diploma holders and 49.2% of them had BSc degree. Among the respondents (61.9%) had work experience of less than 5 years (**See** Table 1**).**

**Table 1:** Socio-demographic characteristic of obstetric caregivers working at labour ward in public health centers of east Gojjam zone, Amhara region, Ethiopia, 2018 G.C. (*N* = 299)

Characteristics	Frequency (n)	Percent (%)
**Age (in years)**		
20–29	194	64.9
30–39	97	32.4
≥ 40	8	2.7
Mean age: 28.96 (± SD = 4.195)		
**Gender**		
Male	162	54.2
Female	137	45.8
**Religion**		
Orthodox	258	86.3
Muslim	30	10
Protestant	10	3.4
Other©	1	0.3
**Profession**		
Health officer	75	25.1
Midwife	93	31.1
Nurse	131	43.8
**Level of education**		
Diploma	149	49.8
BSc degree	147	49.2
Masters	3	1
**Clinical experience (in years)**		
≤ 5	185	61.9
6–9	76	25.4
≥ 10	38	12.7

Other© = Catholic

### Knowledge of study participants about labour pain relief methods

Majority (94.3%) of respondents reported that they knew about labour pain management methods in general, of these 44 (14.7%) knew pharmacologic and 58 (19.4%) knew non-pharmacologic labour pain relief methods only. Nevertheless, 175 (58.5%) of them reported that they knew both labour pain relief methods.

**Fig. 1:**
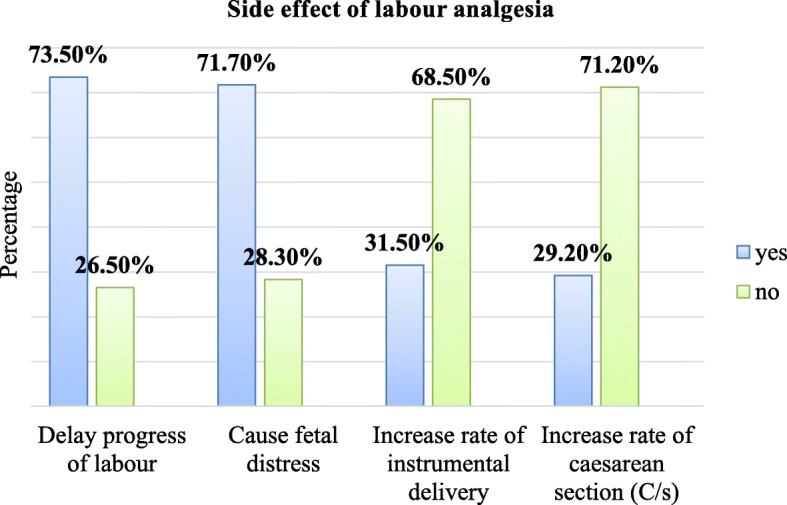
Knowledge of obstetric caregivers on side effect of pharmacologic labour pain relief methods working at labour ward in public health centers of east Gojjam zone, Amhara region, Ethiopia, 2018 (*n* = 219)

Among the study participants who knew pharmacologic methods, 174 (79.5%) of them knew steroidal drugs, 130 (59.4%) systemic opioid’s, 75 (34.2%) epidural analgesia and 24 (11%) inhalational methods, respectively. Of all who knew about pharmacologic labour pain management methods, 161 (73.5%) and 157 (71.7%) of them reported delay progress of labour and fetal distress as a side effect of labour analgesia, respectively (**See** Fig. 1**).**

One hundred fifty-five (51.8%) of respondents reported that they knew a WHO pain ladder. Among the study participants who knew non-pharmacologic labour pain relief methods, psychotherapy 217 (93.1%), allow the mother to ambulate 188 (80.7%), massage the back 175(75.1%), show the woman how to bear down 127 (54.5%) and allow companion of choice of labouring woman 110 (47.2%) were the commonly reported non-pharmacologic labour pain relief methods (**See** Table 2**).**

**Table 2:** Knowledge of obstetric caregivers on Non –pharmacologic labour pain relief methods working at labour ward in public health centers of east Gojjam zone, Amhara region, Ethiopia, 2018 G.C. (*n* = 233)

Types		Frequency (n)	Percent (%)
Psychotherapy	Yes	217	93.1
	No	16	6.9
Allow the mother to ambulate	Yes	188	80.7
	No	45	19.3
Massage the back	Yes	175	75.1
	No	58	24.9
Allow free vertical positioning	Yes	61	26.2
	No	162	73.8
Transcutaneous electrical nerve stimulation	Yes	13	5.6
	No	220	94.4
Show the woman how to bear down	Yes	127	54.5
	No	106	45.5
Acupuncture	Yes	20	8.6
	No	213	91.4
Hypnosis	Yes	13	5.6
	No	220	94.4
Allow companion of labouring woman choice	Yes	110	47.2
	No	123	53.8
Music therapy	Yes	42	18
	No	181	82

In this study, out of the total respondents more than half (54.2%) of obstetric care providers had adequate knowledge on listed types of labour relief methods (with 95% CI = 48.55–59.85%) while the rest 137 (45.8%) of respondents had inadequate knowledge about the listed labour pain relief methods.

### Attitude towards labour pain relief methods

As regards the attitude of obstetric care givers towards labour pain relief methods**,** more than half (57.2%) of them had positive attitude whereas 42.8% of them had negative attitude towards managing of labour pain. The majority (86.3%) of the study participants believed managing of labour could help labouring woman to cope labour pain. However, 55.9% of them thought that pharmacologic labour pain relief method (analgesic) is not required for managing such labour pain.

### Use of labour pain relief methods

The result of this study showed that the overall use of labour pain relief methods among obstetric caregivers was reported as 34.4% (30.4% non-pharmacologic and 8.4% pharmacologic pain relief methods) with 95% confidence interval of [29.01–39.78], respectively. From the non-pharmacologic labour pain management methods, psychotherapy was the most widely used method, which was prescribed by 132 (44.2%) of obstetric caregivers followed by massaging the back 122(40.8%) **(See** Table 3***).***

**Table 3:** Non -pharmacologic methods use by obstetric caregivers working at labour ward in public health centers of east Gojjam zone, Amhara region, Ethiopia, 2018 G.C. (n = 299)

Types	Frequency	(n)	Percent (%)
Psychotherapy	Yes	132	44.1
	No	167	55.9
Allow the mother to ambulate	Yes	116	38.8
	No	183	61.2
Massage the back	Yes	122	40.8
	No	177	59.2
Allow free vertical positioning	Yes	25	8.4
	No	274	91.6
Show the woman how to bear down	Yes	96	32.1
	No	203	67.9
Hot compress	Yes	7	2.3
	No	292	97.7
Allow companion of her choice	Yes	87	29.1
	No	212	70.9

### Personal preference and pain expectation

From the total study participants, most obstetric caregivers (87.6%) expected labour pain as severe pain while (3.4%) of them expected labour pain as moderate pain. Among the study participants, more than half (55.9%) preferred non-pharmacologic methods while 10.7% of them preferred pharmacologic methods to manage labour pain, respectively. Diclofenac (51.2%) was the highest reported preferred pharmacologic method followed by pethdine (34.1%).

### Reasons for non-utilization of labour pain relief methods

**Fig. 2:**
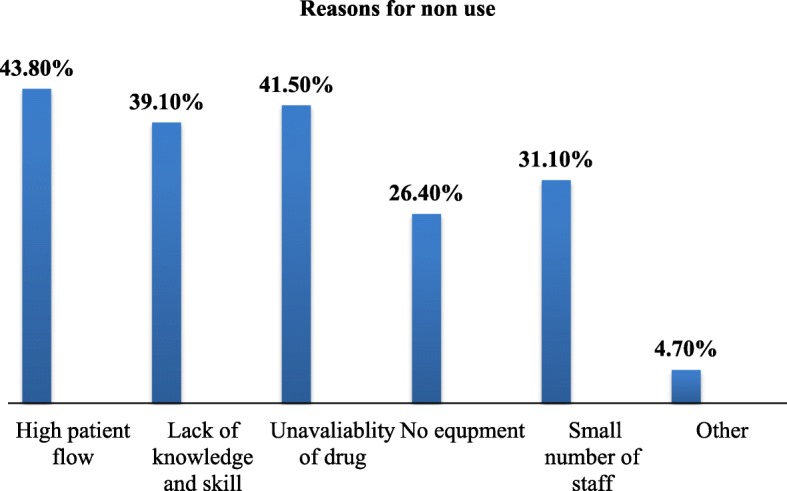
Bar chart showing reasons for non-utilization of labour pain relief methods, 2018. (*n* = 299). Key: Other reasons: Free of side effect and labour pain is a natural process which should not be managed

The most common reasons reported for non-utilization of labour pain relief methods were high patient flow 131 (43.8%) followed by unavailability of drugs 124(41.5%), respectively (See Fig. 2).

Of those who knew pharmacologic methods, 153 (51.2%), 140 (46.8%), 102 (34.1%) and 93 (31.1%) of study participants reported diclofenac, Paracetamol, Pethidine and Hyoscine were available in their health centers for use, respectively. Eighty-seven (29.1%) of study participants reported companion as a choice for labouring woman is not allowed by their health center and 87% of them reported that they didn’t get any special training on labour pain management.

### Factors associated with knowledge of obstetric caregivers towards labour pain relief methods

Profession categories, staff attitude, level of education, and companionship were significantly associated with knowledge of obstetric caregivers about labour pain relief methods in bivariate logistic regression. These variables also remained significantly associated in multivariable logistic regression. Being midwifery professionals were 2.8 times more likely to be knowledgeable about labour pain relief methods than health officer and nurses. [AOR =2.814, 95% CI = (1.574–5.031)]. Professionals with a medium level of education were 3.45 times more likely to be knowledgeable about labour pain relief methods compared to those with a lower level of education [AOR = 3.450, 95% CI = (1.993–5.971)]. Obstetric caregivers who had a positive attitude about labour pain management were 4.37 times more likely to be knowledgeable about labour pain management methods than those who had a negative attitude for labour pain management [AOR = 4.370, 95% CI = (2.523–7.567)] (See Table 4).

**Table 4:** Bivariate and Multivariate analysis of factors associated with knowledge of obstetric caregivers towards labour pain relief methods east Gojjam zone, Amhara regional state, in Ethiopia, 2018. G.C. (n = 299)

	Knowledge of obstetric caregivers		COR (95% CI)	AOR (95% CI)	*P* Value
Characteristics	Adequate	inadequate			
	Frequency (n)	Frequency (n)			
**Profession**					
Midwife	66(71%)	27(29%)	2.801(1.68–4.73)	**2.814(1.574–5.031)**	.**000**
Others	96(46.6%)	110(53.4%)	1.00	1.00	
**Level of education**					
Lower	68 (43.6%)	88(56.4%)	1.00	1.00	
Medium	94(65.7%)	49(34.3%)	2.483(1.55–3.97)	**3.450(1.993–5.971)**	.**000**
**Companion**					
Yes	132 (62.3%)	80(37.7%)	3.135(1.86–5.28)	**2.349(1.314–4.197)**	.**004**
No	30(34.5%)	57 (65.5%)	1.00	1.00	
**Attitude**					
Favorable attitude	118(69%)	53(31%)	4.250(2.61–6.92)	**4.370(2.523–7.567)**	**0.000**
Un Favorable attitude	44(34.4%)	84(65.6%)	1.00	1.00	

Lower level: Diploma; Mid-level: BSc holders; Others: Health officers & Nurses

### Factors associated with use of labour pain relief methods

Bivariate logistic regression revealed that professional categories, knowledge, attitude, experience of obstetric caregivers and allowing a companion for a labouring woman were associated with the use of labour pain relief methods. But only knowledge, attitude and experience of obstetric caregivers remained significantly associated with the use of labour pain relief methods for labouring woman in multivariable logistic regression. Obstetric caregivers who had adequate knowledge about labour pain relief methods for managing labour pain were about 3.82 times more likely to use labour pain relief methods than to those obstetric caregivers who had inadequate knowledge about labour pain relief methods [AOR = 3.821, 95% CI = (2.091–6.980)].

Obstetric caregivers who had a positive attitude for managing labour pain were 2.45 times more likely to use labour pain management methods than those who had a negative attitude for labour pain management [AOR = 2.455, 95% CI = ((1.358–4.436))]. Obstetric caregivers who had an experience of 6–9 years and ≥ 10 years were 2.56 and 2.50 more likely to use labour pain relief methods than those who have ≤5 years’ experience [AOR = 2.56,95% CI = (1.350–4.845) and [AOR = 2.50,95% CI = (1.132–5.524)], respectively (See Table 5).

**Table 5:** Bivariate and Multivariate analysis of factors associated with use of labour pain relief methods among obstetric caregivers east Gojjam zone, Amhara regional state, in Ethiopia, 2018G.C.(n = 299)

	Use of labour pain relief methods		COR (95% CI)	AOR (95% CI)	*P* Value
Variables	Yes	No			
	Frequency (n)	Frequency (n)			
**Profession**					
Midwife	40(44%)	53(56%)	1.713(1.032–2.842)	1.435(.801–2.572)	.225
Others	63(70.4%)	143(29.6%)	1.00	1.00	
**Experience**					
≤ 5 years	52(28.1%)	133(71.9%)	1.00	1.00	
6–9 years	32(42.1%)	44(57.9%)	1.860(1.066–3.246)	**2.56(1.350–4.845)**	.004*
≥ 10 year	19(50%)	19(50%)	2.558(1.255–5.213)	**2.50(1.132–5.524)**	.023*
**Knowledge**					
Inadequate	22(16.1%)	115(83.9%)	1.00	1.00	
Adequate	81(50%)	81(50%)	5.227(3.015–9.063)	**3.82(2.091–6.980)**	.000*
**Attitude**					
Favorable attitude	79(46.2%)	92 (53.8%)	3.721(2.177–6.360)	**2.46(1.358–4.436)**	.000*
Un Favorable attitude	24(18.8%)	104(81.2%)	1.00	1.00	
Companion					
Yes	84(39.6%)	128(60.3%)	2.349(1.317–4.188)	1.458(.761–2.793)	.256
No	19(21.8%)	68(78.2%)	1.00	1.00	

Lower level: Diploma; Mid-level: BSc holders; Others: Health officers & Nurses

## Discussion

The current study aimed to assess knowledge and use of labour pain relief methods and associated factors among obstetric caregivers at public health centers of East Gojjam Zone, Amhara region, Ethiopia**.**

### Knowledge of obstetric caregivers towards labour pain relief methods

In this study, systemic opioid (Pethdine) was reportedly one of the most identified pharmacological pain relief methods, which is similar with studies from Zaria, Greek, and Ibadan (Nigeria), respectively [6, 16, 17]. This might be due to accessibility and low cost of the drug on the market. The present study showed that 45.8% of obstetric caregivers had inadequate knowledge about labour pain relief methods. Our finding is lower than the findings reported from Tigray, Ethiopia (60.1%) and Ibadan, Nigeria (66.7%), respectively [13, 17]. This difference might be explained in terms of difference in study setting and socio- demographic characteristics of study participants. The study also found that midwifery professionals had adequate knowledge than health officer and nurses, which was similar with Australian studies undertaken by Lee et al (2012) [18], which reported knowledge as a significant factor for obstetric analgesia use. In our study, professionals with a medium level of education were 3.4 times more likely to be knowledgeable than those with a lower level of education regarding labour pain relief methods. This might be explained in terms of variation in curriculum content of obstetric courses delivered to health professions based on their level of training programs. Positive attitude of staff also showed significant association with knowledge with regard to labour pain relief methods.

### Use of labour pain relief methods among obstetric caregivers

The current study showed that the overall use of labour pain relief methods among obstetric caregivers was reported to be 34.4% (30.4% non-pharmacological and 8.4% pharmacological) methods, respectively. This finding is inconsistent with earlier studies’ findings from different parts of Ethiopia: Tigray, 43.3% [13], Addis Ababa, 47.5% [19] and Amhara, 40.1% [14]. The reasons might be the preceding studies were conducted in public hospitals where better knowledge of labour pain relief methods and drug availability are potentially high.

In our study, the use of non-pharmacological methods was consistent with studies done in Dhaka, Bangladesh and Ghana where allowing laboring woman to move freely, showing the patient how to bear down, allowing companion, and massaging the back were the most applied non-pharmacologic pain relief methods [20, 21]. This study found that the use of pharmacologic labour pain relief method by obstetric caregivers was reported to be 8.4% of which pethidine, diclofenac, paracetamol and Hyoscine were mostly used. This result is consistent with studies done in Bangladesh and Ghana, where these drugs were also used as pharmacological labour pain relief methods [20, 21].

On the other hand, this result is higher than the findings from studies done in Amhara region referral hospital [14] and Tigray region general hospital [13]. This might be due to time difference related to previous studies and increased awareness of obstetric caregivers towards labour pain management through time. Nonetheless, this result is found to be lower than the findings from Nigeria, 49% [3], Kenya, 18% [8] and Addis Ababa Ethiopia, 54.2% [19], respectively.

This study revealed that obstetric caregivers who had a positive attitude for managing labor pain were 2.45 times more likely to use labor pain management methods than those who had a negative attitude for labour pain management [AOR = 2.455, 95% CI = ((1.358–4.436))]. This finding is consistent with a study done in Bangladesh [20], and Ethiopia [15], respectively.

In this study, obstetric caregivers who had adequate knowledge about labour pain relief methods for managing labour pain were 3.82 times more likely to use labour pain relief methods than those who had inadequate knowledge about labour pain relief methods [AOR = 3.821, 95% CI = (2.091–6.980)], which is inconsistent with studies from Nigeria and Abha Maternity Hospital in Saudi Arabia where health care providers who had adequate knowledge were more likely to provide labour relief method for labouring woman [17, 22].

The current study reported that obstetric caregivers who had an experience of 6–9 and ≥ 10 years were more likely to use labour pain relief methods than those who had ≤5 years’ experience [AOR = 2.56,95% CI = (1.350–4.845) and [AOR = 2.50,95% CI = (1.132–5.524), respectively. This finding is similar to a study done in the U.S where more experienced nurses provide more labour support [23]. In this study, high patient flow, small number of staff, lack of knowledge, limited skill and unavailability of equipment and drugs for managing labour pain were factors affecting the use of labour pain relief methods. This finding is also consistent with a study done in Tigray region general hospitals, Ethiopia [13], Amhara region referral hospitals, [14], Addis Ababa, Ethiopia [19], Zaria, Nigeria [6] and Saudi Arabia [22].

### Limitation of the study

The results of this study must be interpreted in the light of the following limitations. The study was conducted in public health centers of Amhara Region, Ethiopia. The perspectives of health providers in private health facilities were not explored in the study. The findings of this study are thus mainly applied to obstetric care providers in the study setting. Since the study was cross sectional study, it did not address the cause and effect relationship of the factors and the outcome variables.

## Conclusion

Although labor pain management is accepted and implemented in many countries of the world, pain management during labor is not often practiced. This study is essential as it aims to assess knowledge, and use of labour pain relief methods and associated factors among obstetric caregivers in this study area of Ethiopia.

The current study revealed that the overall use of labour pain by obstetric caregivers was low. Systemic opioid (Pethdine) was reportedly one of the most known pharmacological pain relief methods in this study. Providers’ knowledge, attitude and work experience had shown statistical significance with the use of labour pain relief methods. Task-oriented in- service training is thus required to fortify obstetric caregivers’ knowledge and attitude towards the use of labour pain relief methods. Regular supervision of obstetric caregivers and logistic supplies and analgesic drugs are also needed for effective labour pain management. Furthermore, researchers in the field are recommended to examine the use of labour pain relief methods from maternal’ request point of view.

Overall, this is a fascinating study which has the potential to provide cross cultural education of caregivers from a high income countries who might be caring for immigrant women from low income countries, and may also be a very useful reference for planners of obstetric and midwifery care and education in low income countries.

## Data Availability

All the data included in the manuscript can be accessed from the corresponding author with an email address keralemante2010@gmail.com

## Abbreviations

AOR: Adjusted Odds Ratio
CI: Confidence Interval
COR: Crude Odd Ratio
HCP: Health Care Providers
OCGs: Obstetric Caregivers
SPSS: Statistical Package for Social Sciences
